# Prévalence de la dengue chez les patients présentant un syndrome fébrile au Centre hospitalier universitaire Sylvanus Olympio de Lomé (Togo) en 2017

**DOI:** 10.48327/mtsi.2021.183

**Published:** 2021-12-09

**Authors:** Mounerou SALOU, Wendpouiré Ida Carine ZIDA-COMPAORÉ, Fifonsi Adjidossi GBEASOR-KOMLANVI, Messan FOLLY-GBOGBOE, Abla Ahouefa KONOU, Sika DOSSIM, Zouberou MAMA, DOUFFAN M., Didier Koumavi EKOUEVI, Anoumou Y. DAGNRA

**Affiliations:** 1 Faculté des sciences de la santé (FSS), Département des sciences pharmaceutiques, Laboratoire de biologie et d'immunologie (BIOLIM), Département des sciences fondamentales, Université de Lomé, Togo; 2 Faculté des sciences de la santé (FSS), Département de santé publique, Université de Lomé, Togo

**Keywords:** Prévalence, Dengue, Paludisme, Sérologie, TDR, Hôpital, Togo, Afrique subsaharienne, Prevalence, Dengue, Malaria, Serology, RDT, Hospital, Togo, Sub-Saharan Africa

## Abstract

**Objectif:**

Estimer la prévalence de la dengue chez les patients présentant un syndrome fébrile au Centre hospitalier universitaire Sylvanus Olympio de Lomé.

**Méthode:**

Étude transversale à visée descriptive.

**Résultats:**

Cent quarante-sept patients ayant un âge médian de 36 ans, intervalle interquartile: [23,5-51,5], ont été inclus dans l’étude. La prévalence du paludisme dans l’échantillon était de 10,2 % (IC95%: [5,8-16,3]) et celle de la dengue par ELISA était de 17 % (IC95%: [11,3-24,1]). Le pourcentage de concordance globale entre le test de diagnostic rapide (TDR) et le test ELISA était de 80,9 %, (IC95%: [73,7-86,9]). Le pourcentage de concordance positive entre le TDR Dengue et le test ELISA Dengue était de 36 % (IC95%: [17,9-57,5]) alors que le pourcentage de concordance négative était de 90,2 % (IC95%: [83,4-94,8]).

**Conclusion:**

Cette étude montre que la dengue est, au même titre que le paludisme, responsable de syndromes fébriles et qu'elle est présente au Togo.

## Abstract (en)

**Aims:**

Dengue is the most common arbovirus in the world. In Africa, dengue virus is endemic in almost every country; however, in Togo few data are available. The aim of this study was to estimate the prevalence of dengue fever among patients with febrile syndrome at the Centre hospitalier universitaire Sylvanus Olympio of Lomé. **Procedure.** A cross sectional study was conducted in the Centre hospitalier universitaire Sylvanus Olympio of Lomé.

**Results:**

One hundred forty-seven patients with a median age of 36 years, interquartile range: [23.5-51.5], were included in the study. The prevalence of malaria in the sample was 10.2% (95% CI: [5.8-16.3]) and the prevalence of dengue fever by ELISA was 17% (95% CI: [11.3-24.1]). The overall percent agreement between the RDT Dengue NS1 and ELISA for dengue was 80.9% (95% CI: [73.7-86.9]). The positive percent agreement (PPA) between the RDT assay and the ELISA assay considered as the reference was 36%, 95% CI: [17.9-57.5]), while the negative percent agreement (NPA) between the two assays was 90.2% (95% CI: [83.4-94.8]).

**Conclusion:**

This study shows that dengue is as much as malaria responsible of febrile syndromes and that it is present in Togo.

## Introduction

La dengue est l'une des 17 maladies tropicales négligées (MTN) figurant sur la feuille de route de l'Organisation mondiale de la santé (OMS) [13]. C'est l'arbovirose la plus répandue dans le monde. Elle est présente sous les tropiques avec des variations locales de risque qui sont fonction des précipitations, de la température et de l'urbanisation rapide et non maîtrisée. Elle sévit dans les régions tropicales et subtropicales du monde entier, avec une prédilection pour les zones urbaines et semi-urbaines [15, 27]. Il existe quatre sérotypes du virus de la dengue notés DENV-1 à DENV-4 et qui sont retrouvés dans plusieurs pays de l'Afrique subsaharienne [10, 21].

La dengue est une infection virale transmise à l'homme par la piqûre des femelles de moustiques du genre Aedes infectées par le virus. Les piqûres ont lieu tôt le matin et le soir avant le crépuscule. Les sujets infectés par le virus de la dengue peuvent transmettre l'infection pendant 4 à 5 jours et au maximum 12 jours [15]. Cette infection provoque un syndrome de type grippal qui peut évoluer vers des complications allant jusqu’à des formes de dengue sévère, potentiellement mortelles [15].

L'incidence mondiale de la dengue a progressé de manière spectaculaire au cours des dernières décennies. Environ la moitié de la population est exposée au risque et l'année 2016 a été marquée par d'importantes flambées de dengue dans le monde entier [15]. Selon des estimations récentes, 390 millions de cas de dengue sont enregistrés chaque année [4], et 3,9 milliards de personnes vivant dans 128 pays sont exposées à l'infection par les virus de la dengue [7]. Le nombre de cas varie d'une région à l'autre: en Europe en 2018, 27 pays ont rapporté 2 191 cas dont 2 033 confirmés avec un pic observé au mois de novembre [9]. La région OMS des Amériques a notifié plus de 2,38 millions de cas en 2016 avec 1 032 décès. La région du Pacifique occidental a signalé plus de 375 000 cas suspects de dengue au cours de la même année dont 176 411 notifiés aux Philippines et 100 028 en Malaisie. Dans la région africaine, le Burkina Faso a notifié en 2016 une flambée localisée avec 1 061 cas probables [15].

En Afrique, le virus de la dengue est endémique dans presque tous les pays. Cependant, cette maladie reste mal connue et sous-déclarée en raison de la faible sensibilisation de la part des prestataires de soins de santé, du poids endémique d'autres maladies fébriles, de la non-disponibilité des tests pour le diagnostic biologique et de l'absence d'une surveillance systématique [2].

Dans le cadre de la lutte contre la dengue, l'OMS avait adopté en 2012 une stratégie globale dans le but d'en réduire la charge morbide en fixant trois objectifs spécifiques: i) réduire la mortalité d'au moins 50 % entre 2010 et 2020; ii) réduire la morbidité de la dengue d'au moins 25 % entre 2010 et 2020; iii) et estimer le fardeau réel de la maladie à l'horizon de l'année 2015 [12].

Cependant, le nombre de cas de dengue notifiés à l'OMS a été multiplié par plus de 8 au cours des deux dernières décennies, passant de 505 430 cas en 2000 à plus de 2,4 millions en 2010 et à 4,2 millions en 2019 tandis que le nombre de décès déclarés entre 2000 et 2015 est passé de 960 à 4 032 [15].

Le Togo est un pays d'Afrique subsaharienne avec deux types de climats: un climat tropical au nord caractérisé par une saison sèche et une saison pluvieuse et un climat subéquatorial au sud caractérisé par deux saisons sèches et deux saisons pluvieuses. Ces conditions climatiques favorisent la prolifération des moustiques [14] et, par conséquent, les maladies dont ils sont vecteurs. Au Togo, peu de données sont disponibles sur la dengue. Une étude réalisée en 2012 décrivant la situation de cette maladie en Afrique a rapporté que le Togo fait partie des pays déclarant les cas de dengue depuis 2000 [26].

Face à un tel manque de données, cette étude pilote a été initiée dans le but de contribuer à l'analyse des étiologies des fièvres non palustres au Togo. Son objectif était d'estimer la prévalence de la dengue chez les patients présentant un syndrome fébrile au Centre hospitalier universitaire Sylvanus Olympio (CHU SO) de Lomé.

## Méthodes

### Schéma et période d’étude

Il s'agissait d'une étude transversale à visée descriptive qui s'est déroulée du 1er mai au 31 août 2017 à Lomé dans les services d'urgence médicale, de pédiatrie, de maladies infectieuses, de médecine interne et de réanimation polyvalente du CHU SO. Le choix de cet établissement s'est fait sur son statut d'hôpital de référence dans la pyramide sanitaire du Togo.

La population cible de cette étude était les habitants de la ville de Lomé et la population source était constituée par les malades présents sur le site pendant la période d’étude.

Critères d'inclusion:

• être un patient du CHU SO quels que soient l’âge et le sexe;
• présenter une fièvre avec une température corporelle supérieure ou égale à 38 °C;
• donner son accord verbal pour participer à l’étude ou obtenir celui du parent/tuteur pour les mineurs.

La prise de température était axillaire.

Critère de non-inclusion: absence de prélèvement sanguin.

### Échantillonnage

L’échantillonnage de la population a été exhaustif: tous les patients répondant aux critères de sélection ont été enrôlés.

### Collecte des données: questionnaire, prélèvements, tests

Un questionnaire standardisé a été utilisé pour collecter les caractéristiques sociodémographiques et cliniques de chaque patient.

Deux types de prélèvements ont été réalisés pour les analyses biologiques. Un prélèvement veineux de 5 ml de sang au pli du coude dans un tube EDTA qui a servi à la réalisation du test ELISA pour la recherche de la dengue, et un prélèvement capillaire sur le majeur/talon (pour les bébés) qui a permis de réaliser le diagnostic par test de diagnostic rapide (TDR) de la dengue et par goutte épaisse pour le paludisme.

Les tests TDR ont été réalisés immédiatement après le prélèvement et pour le test ELISA de la dengue des aliquotes de plasma ont été réalisés et conservés entre 2 et 8 °C en attendant la fin de l'enquête pour les analyses de laboratoire.

### Dépistage du paludisme et de la dengue

Les tests utilisés pour le dépistage du paludisme étaient la goutte épaisse (GE) et le frottis sanguin sur lame colorés au Giemsa. Le test a permis de déterminer l'espèce plasmodiale ainsi que la parasitémie (nombre de trophozoites pour 100 leucocytes). Les patients présentant une GE positive et un frottis négatif ont été considérés comme des cas positifs.

Pour le dépistage de la dengue, deux tests ont été effectués, un TDR à partir du sang capillaire et un test ELISA à partir du plasma. Il s'agissait du TDR ADVANTAGE DENGUE NS1 AG CARD (Mitra & Co. Pvt. Ltd, Inde) pour la détection de l'antigène NS1 Ag de la dengue dans le sérum/plasma humain (en abrégé Dengue NS1 Ag Card^®^) et du test ELISA Dengue NS1 Ag MICROLISA^®^ (Mitra & Co. Pvt. Ltd, New Delhi, Inde).

Les tests ont été réalisés au Laboratoire de biologie moléculaire et d'immunologie (BIOLIM) de la Faculté des sciences de la santé de l'Université de Lomé. Tous les tests et l'interprétation des résultats ont été effectués conformément aux instructions du fabricant.

### Définition des critères de performance du test TDR

En l'absence d'un test de référence pour le diagnostic de la dengue au Togo, par exemple un test virologique, une détection d'ARN, une séroconversion en IgM ou IgG, ou une augmentation (x 4) des taux d'IgG [19], il n'a pas été possible d’évaluer la sensibilité et la spécificité du test TDR. En revanche, nous avons réalisé une comparaison de méthodes, avec calcul des pourcentages de concordance positive, concordance négative et concordance globale entre le test TDR et le test ELISA pris comme technique « de référence ».

### Traitement des données

Les données de l’étude ont été saisies sous Epidata 3.1 et analysées dans R version 1.3.959. Les analyses statistiques ont permis de présenter les prévalences sous forme de proportion avec leur intervalle de confiance à 95 % (IC95 %).

## Résultats

Au total, 147 patients ont été inclus dans l’étude.

### Caractéristiques sociodémographiques et cliniques

L’âge médian des patients était de 36 ans, intervalle interquartile (IIQ): [23,5-51,5]. Le sex-ratio homme/femme était de 1,01 et 50,3 % des patients étaient mariés ou en union libre (Tableau I).

**Tableau I:** Caractéristiques sociodémographiques et cliniques (N = 147) Sociodemographic and clinical characteristics (N = 147)

	**n**	**%**
**Sexe**		
masculin	74	50,3
féminin	73	49,7
**Âge (années)**		
< 18	13	8,8
18-50	90	61,2
≥ 50	44	30,0
**Situation matrimoniale**		
célibataire / divorcé(e)	52	35,5
marié(e)/ union libre	74	50,3
veuf(ve)	8	5,4
non applicable^†^	13	8,8
**Recherche** *P. falciparum*		
négatif	132	89,8
positif	15	10,2
**TDR Dengue**		
négatif	126	85,7
positif	21	14,3
**ELISA Dengue**		
négatif	122	83,0
positif	25	17,0

† enfants

TDR: test de diagnostic rapide

### Prévalence du paludisme et de la dengue

La prévalence de la dengue telle qu'observée en utilisant le test TDR et le test ELISA était respectivement de 14,3 % (IC95%: [9,1-21,0]) et de 17 % (IC95%: [11,3-24,1]).

La séroprévalence de la dengue observée par le test TDR était de 15,1 % et de 13,5 % respectivement chez les femmes et chez les hommes (p = 0,973), et la séroprévalence observée par ELISA était de 20,3 % et 13,7 % chez les hommes et chez les femmes respectivement (p = 0,400) (Tableau II).

La prévalence de la dengue était plus élevée chez les patients de 18 à 50 ans pour le TDR (p = 0,233) et plus élevée chez les moins de 18 ans pour le test par ELISA (p = 0,250) (Tableau II).

**Tableau II:** Prévalence de la dengue selon le sexe et selon l’âge Prevalence of dengue according to sex and age

		TDR Dengue				ELISA Dengue			
	N	THR +	Prévalence (%)	IC à 95 %	p-value	ELISA +	Prévalence (%)	IC à 95 %	p-value
**Sexe**					0,973			0,400
masculin	74	10	13,5	[6,7-23,4]		15	20,3	[11,8-31,2]	
féminin	73	11	15,1	[7,8-25,4]		10	13,7	[6,8-23,7]	
**Âge (années)**					0,233		0,250
< 18	13	2	15,4	[1,9-4,5]		4	30,8	[9,1-61,4]	
18-50	90	16	17,8	[10,5-27,2]		16	17,8	[10,5-27,2]	
≥ 50	44	3	6,8	[1,4-18,7]		5	11,4	[3,8-24,5]	

IC95%: intervalle de confiance à 95 %; TDR: test de diagnostic rapide; +: positivité du test

La prévalence du paludisme dans l’échantillon était de 10,2 % (IC95%: [5,8-16,3]).

### Co-infection paludisme et dengue

La co-infection était présente dans cette étude. Par TDR Dengue, 3 patients avaient une co-infection paludisme-dengue tandis que par le test ELISA, 5 patients étaient porteurs à la fois du virus de la dengue et du *P. falciparum.*

Les cas de co-infection paludisme-dengue étaient des adultes âgés de 17, 47 et 43 ans pour le TDR Dengue et de 16, 17, 20, 32 et 79 ans pour le test ELISA Dengue. Parmi les 3 positifs en TDR, seul 1 était également positif au test ELISA.

### Pourcentage de concordance globale entre les tests utilisés dans le dépistage de la dengue

Le pourcentage de concordance globale entre le TDR Dengue et le test ELISA Dengue était de 80,9 % (IC95%: [73,7-86,9]) (Tableau III).

**Tableau III:** Pourcentage de concordance globale entre le TDR (Dengue NS1 Ag Card^®^) et le test ELISA dans le dépistage de la dengue Overall percent agreement between the RDT (Dengue NS1 Ag Card^®^) and ELISA in dengue screening

THR	ELISA	Total
	positive	négative	
**Positive**	9	12	21
**Négative**	16	110	126
**Total**	**25**	**122**	**147**

### Pourcentage de concordance positive

En prenant le test ELISA comme technique dite de référence, le pourcentage de concordance positive du test TDR vis-à-vis du test ELISA était 36 % [9/(9+16)] avec un IC95%: [17,9-57,5].

### Pourcentage de concordance négative

En prenant le test ELISA comme technique dite de référence, le pourcentage de concordance négative du test TDR vis-à-vis du test ELISA était 90,2 % [110/(12+110)] avec un IC95%: [83,4-94,8].

## Discussion

### Prévalence de la dengue et du paludisme

La prévalence observée de la dengue dans notre étude était de 17 % tandis que la séroprévalence de l'antigène NS1 spécifique de la dengue retrouvée dans l’étude d'Oyero et Ayukekbong [17] au Nigéria était deux fois supérieure. La détection de l'antigène NS1 de la dengue suggère une dengue aiguë chez les patients de l’étude, car il a été démontré que sa présence était détectée dans le sérum dans les 24 heures et jusqu’à 9 jours suivant l'apparition des symptômes [25]. En Nouvelle-Guinée, une prévalence plus faible de 8 % a été retrouvée [22].Selon une méta-analyse réalisée par Simo et al, la prévalence de la dengue en Afrique variait entre 8,4 % et 24,8 % chez les populations présentant de la fièvre [23]. Ces disparités pourraient s'expliquer par la période (saison) de l'enquête d'une part, et d'autre part du fait que dans notre contexte de travail, la majorité des consultations étaient des références vers le CHU.

Au Togo, le paludisme est endémique avec une transmission qui dure toute l'année sur l'ensemble du territoire national. Toute la population togolaise est exposée au risque de paludisme [11]. Selon le Programme national de lutte contre le paludisme (PNLP), cette maladie représentait 26,4 % des motifs de consultation et d'hospitalisation en 2015. Au cours de la même année, 404 253 cas de paludisme ont été enregistrés chez les enfants de moins de 5 ans et 50 249 chez les femmes enceintes [20]. Dans notre étude, le paludisme était présent chez 10,2 % des patients inclus: ce faible taux pourrait s'expliquer par la réussite des programmes de prévention mis en place par le ministère en charge de la santé. Notre prévalence était également plus faible que celle de l’étude d'Ouangré et al au Burkina Faso [16]. Cette faible prévalence du paludisme par rapport à celle de la dengue révèle l'urgente nécessité de trouver un TDR au coût abordable pour le dépistage de la dengue en routine hospitalière, permettant une meilleure prise en charge des fièvres non palustres, afin d’éviter le traitement abusif par antipaludéens des patients avec GE négative et de limiter le risque de résistance aux antipaludéens.

### Performances du TDR Dengue NS1 Ag Card^®^

Les performances des méthodes disponibles pour le diagnostic de la dengue dépendent principalement de la période de la maladie. L'ARN viral de la dengue et les antigènes (Ag) NS1 solubles sont détectables pendant la phase aiguë précoce de l'infection, si bien que la sensibilité de l'isolement du virus, de la qReverse-Transcription-PCR et de la détection des antigènes NS1 est plus élevée les premiers jours de la maladie [24].

La détection de la protéine NS1 peut permettre de diagnostiquer l'infection, et sa circulation dans le sang des patients pendant la phase clinique de la maladie suggère une contribution de la protéine non structurelle aux pathogènes du virus de la dengue [1]. Le TDR Ag NS1 utilisé dans notre étude avait une faible concordance positive avec le test ELISA pris comme référence (36 %) mais une bonne concordance négative (90 %). Nos résultats sont comparables à ceux de Pal et al qui ont évalué dans leur étude les TDR de l'Ag NS1 de la dengue et les kits ELISA [18]. Selon les mêmes auteurs, la sensibilité des tests NS1 dans la littérature peut varier en fonction de la conception de l’étude et de la méthode de référence utilisée, de 58 à 99 % pour les TDR et de 37 à 93 % pour les ELISA. En outre, les TDR ont une sensibilité plus faible que les ELISA, par conséquent la valeur prédictive négative d'un ELISA NS1 est probablement supérieure à celle des TDR. La conservation des tests influe également sur la qualité des résultats. En effet, une étude de stabilité thermique a démontré une perte de performance de certains TDR lorsqu'ils étaient stockés à une température ambiante élevée pendant 3 mois [6].

Selon Dussart et al, l'utilisation de l'antigène NS1 comme test de première ligne pour le diagnostic de la dengue, à l'aide d’échantillons prélevés au moment de l'apparition des symptômes cliniques, pourrait contribuer à accélérer le diagnostic de la dengue dans les laboratoires de diagnostic clinique, à condition d'employer des tests ayant de bonnes performances; en outre, l'utilisation d'une combinaison de MAC-ELISA et de détection de l'antigène NS1 pourrait augmenter la sensibilité clinique globale des deux tests [8]. Dans une étude réalisée en Indonésie, la performance du test de l'antigène NS1 a été affectée par le statut infectieux des patients et l'origine géographique des échantillons [3].

Notre étude est une première au Togo, mais elle présente certaines limites:

• la taille réduite de l’échantillon qui ne reflète pas la population générale peut entraîner une sur/sous-estimation de la prévalence de la dengue et du paludisme;
• le lieu de résidence des patients n'a pas été renseigné, ce qui empêche la recherche de lien avec la prévalence des maladies étudiées;
• l’étude a été conduite en pleine saison de pluies, ne permettant pas d'estimer la prévalence à différentes périodes de l'année;
• les différents sérotypes de dengue circulant au Togo n'ont pas été identifiés.

## Conclusion

Cette étude montre que la dengue est, au même titre que le paludisme, responsable de syndromes fébriles et qu'elle est présente au Togo. L’étude suggère également une possibilité de co-infection entre le paludisme et la dengue. Cependant, aucun diagnostic de routine n'est réalisé par les professionnels de santé en cas de suspicion de fièvre non palustre: l'utilisation de TDR pour la détection de façon conjointe de l'antigène NS1 et des anticorps IgM, en première intention, permettrait de prendre en charge précocement les cas.

## Remerciements

Nous remercions le laboratoire EDIMAMEL pour l'octroi des réactifs pour le test ELISA ainsi que des tests de diagnostic rapide (TDR) pour le diagnostic de la dengue.

## Lien d'intérêts

Les auteurs ne déclarent aucun lien d'intérêt.

## Contribution des auteurs

MS, AYD et DKE ont rédigé le protocole de l’étude.

MS, WICZC, MFG ont conduit les enquêtes et réalisé les manipulations de laboratoire.

FAGK, AAK et SD ont réalisé la prospection bibliographique.

ZM et MD ont réalisé les analyses statistiques.

MS et WICZC ont interprété les résultats et rédigé le premier draft du manuscrit.

Tous les auteurs ont relu et validé le manuscrit.
